# Does PGT-A improve assisted reproduction treatment success rates: what can the UK Register data tell us?

**DOI:** 10.1007/s10815-022-02612-y

**Published:** 2022-09-21

**Authors:** Stephen A. Roberts, Jack Wilkinson, Andy Vail, Daniel R. Brison

**Affiliations:** 1 Centre for Biostatistics, University of Manchester, Manchester Academic Health Science Centre, Manchester, M13 9PL UK; 2 Department of Reproductive Medicine, Old St Mary’s Hospital, Manchester University Hospitals NHS Foundation Trust, Manchester Academic Health Science Centre, Oxford Road, Manchester, M13 9WL UK

**Keywords:** Preimplantation genetic testing aneuploidy, Retrospective study, Registry data, Assisted Reproduction, Live birth rates

## Abstract

**Purpose:**

To show how naïve analyses of aggregated UK ART Register data held by the Human Fertilisation and Embryology Authority to estimate the effects of PGT-A can be severely misleading and to indicate how it may be possible to do a more credible analysis. Given the limitations of the Register, we consider the extent to which such an analysis has the potential to answer questions about the real-world effectiveness of PGT-A.

**Methods:**

We utilise the publicly available Register datasets and construct logistic regression models for live birth events (LBE) which adjust for confounding. We compare all PGT-A cycles to control groups of cycles that could have had PGT-A, excluding cycles that did not progress to having embryos for biopsy.

**Results:**

The primary model gives an odds ratio for LBE of 0.82 (95% *CI* 0.68–1.00) suggesting PGT-A may be detrimental rather than beneficial. However, due to limitations in the availability of important variables in the public dataset, this cannot be considered a definitive estimate. We outline the steps required to enable a credible analysis of the Register data.

**Conclusion:**

If we compare like with like groups, we obtain estimates of the effect of PGT-A that suggest an overall modest reduction in treatment success rates. These are in direct contrast to an invalid comparison of crude success rates. A detailed analysis of a fuller dataset is warranted, but it remains to be demonstrated whether the UK Register data can provide useful estimates of the impact of PGT-A when used as a treatment add-on.

**Supplementary Information:**

The online version contains supplementary material available at 10.1007/s10815-022-02612-y.

## Background

Preimplantation genetic screening for aneuploidy (PGT-A) covers a variety of procedures for detecting non-diploid embryos post culture but prior to implantation during an IVF treatment cycle. The rationale is that some aneuploid embryos are non-viable, and therefore selection of non-aneuploid embryos will improve success rates, a notion which has been challenged on several grounds [1] including the presence of mosaicism in the embryos [2] and the potential for an embryo to correct such errors [3, 4]. A number of techniques, mainly requiring invasive biopsy of the embryo, have been employed [5] with whole genome sequencing of trophectoderm biopsies from blastocyst-stage embryos being the current standard practice The risks and benefits of PGT-A have been highly controversial with strong commercial interests influencing the debate [6–8].

There have been a number of randomised controlled trials addressing different scenarios for the use of PGT-A with different PGT-A techniques and different endpoints, many of which have been small and of poor quality [9]. Meta-analyses suggest PGT-A is not of proven effectiveness in terms of live birth rates, and older versions appear to have been detrimental overall [9]. Three recent trials using next-generation sequencing from blastocyst biopsies with cryopreservation all show small effect sizes favouring the control arms: *OR* 0.93 (0.69–1.3) [10]; *OR* 0.91 (0.51 to 1.63) [11]; *OR* = 0.75 (0.57 to 1.0) [12], the latter being a cumulative live birth outcome. In the UK, the Human Fertilisation and Embryology Authority (HFEA) has reviewed the evidence as part of their “traffic light” evaluation of IVF add-ons and concluded that PGT-A should have a “red” rating — “No evidence to show that it is effective and safe” [13].

As the UK regulator, the HFEA maintains a register of all ART treatments and their outcomes. This includes the use of PGT-A in each treatment cycle. The HFEA retains a full dataset, extracts of which are made available to bona fide researchers on application and subject to strict confidentiality restrictions. They also maintain publicly accessible datasets with a limited, highly anonymised, subset of the data which can be downloaded from their website [14]. Additionally, as a public body, it is subject to the UK Freedom of Information (FoI) legislation and so responds to legitimate and reasonable requests for summary information. Superficially, it may be appealing to utilise these ‘real world’ practice data to investigate the utility of PGT-A, although it is not clear whether the available data would enable a valid analysis.

Such a FoI request was recently used to obtain crude success rates for PGT-A and non-PGT-A treatment cycles and these data have been cited in publications, conferences and webinars [15], including a paper in this journal [16] to suggest that, despite the evidence from RCTs, PGT-A is an effective treatment add-on for IVF in clinical practice. The data used consisted solely of aggregated numbers of cycles, embryos transferred and live birth events (LBE) for 6 age bands over a 3-year period (2016–8). They are reproduced in the supplementary material (Table S1). Such an analysis is fundamentally flawed and potentially seriously misleading because:

1. The use of crude aggregate rates provides no contextual information and no ability to compare like with like. The FoI supplied data are not well defined in terms of inclusions and exclusions.
2. There was no adjustment for confounding except for banding by age group: this is particularly problematic when the treatment is only offered by selected clinics and is dependent on patient choice and ability to pay. Clinics offering PGT-A have potentially different patient demographics, treatment protocols and pathways as well as differing treatment eligibility criteria.
3. A comparison group of all non-PGT-A cycles includes many cycles that would likely not have had PGT-A, as there were insufficient embryos to biopsy. As the Register only records PGT-A treatments that were delivered, the comparator group must consist of treatments that could have had PGT-A if the option were available.

## Aims

In this paper, we aim to show how naïve analyses of aggregated register data to estimate the effects of PGT-A can be severely misleading. We will utilise the publicly available HFEA Register data to determine if the observational data could in fact be compatible with the results of RCTs despite the serious limitations of these data.

Having looked at the publicly available data, we then aim to indicate how it may be possible to do a more epidemiologically credible analysis of the full HFEA Register data. Given the limitations of the register data, we consider the extent to which such an analysis has the potential to answer questions about the real-world effectiveness of PGT-A.

## Methods

### Dataset

We utilised the data publicly available for research on the HFEA website [14] and the 2015–6 and 2017–8 cohorts were downloaded on 27/4/2022. After minimal reformatting to harmonise variable names and coding, these were merged into a single dataset and the subset of IVF treatments commencing in 2016–2018 extracted.

From this dataset we extracted all IVF cycles. Cycles using donated eggs and PGT-M cycles were excluded. We included only cycles where the recorded intention was to proceed to implantation; this excludes treatments whose sole purpose was to create eggs or embryos for storage or donation.

These inclusion/exclusion criteria approximate to those used in creating the data provided for FoI. They differ somewhat due to differing definitions used by the HFEA for FoI requests and those provided in the public research database.

Additionally, we excluded 17 cycles with missing age, 11 fresh transfers with missing data on IVF or ICSI, 68 that were not identified as either fresh or frozen cycles and 55 recorded as both a fresh and a frozen cycle.

### PGT-A cycles

All cycles recorded as using PGT-A were included.

### Control cycles

PGT-A requires that the treatment proceeds as far as producing embryos for biopsy and the Register records embryo biopsies, not the intention to perform such. As PGT-A is used for embryo selection, it usually also requires that there be more than one embryo available. Therefore, PGT-A treatment cycles must be compared to non-PGT-A (control) cycles that could at least potentially have had PGT-A, if it were available. That is, we want a control group of cycles that progressed as far as having viable embryos after embryo culture that could have been biopsied and exclude cycles that failed to progress to that stage. Unfortunately, such detailed intermediate outcomes are not available in the Register, and we have to use surrogate variables to approximate to this restriction. We defined three such groups:

1. From the outcomes available, the subset of cycles that had an embryo transferred and also had an embryo stored (cryopreserved) for future storage do form a subset which could potentially have had PGT-A (such cycles must have had at least 2 assessable embryos at the time of transfer). We also limit this to fresh cycles, as this represents current practice at the time most treatments involved a primary transfer of 1 or 2 fresh embryos with frozen transfers being secondary. As the public data do not link fresh and frozen transfers, consideration of the frozen cycles was not possible using these definitions. Thus we defined a primary control group as having:
  $$\text{Embryos.Transferred > 0 and Embryos.Stored > 0 and Fresh}$$

This definition has the disadvantage that it excludes patients treated in centres where embryo storage is not available or in private centres where patients cannot, or choose not, to pay for storage.

2. As an alternative, we considered simply limiting the control cycles to those where a reasonable number of embryos were available for selection. As the data available were banded (lowest band pools 1–5 embryos), we selected those with > 5 embryos created. Thus, we define a secondary control group with: 
  $$\text{Embryos.Created > 5 and Fresh}$$

Data﻿ suggest t﻿ha﻿t between 30 and 50% of embryos created will survive to the blastocyst stage, depending on the quality threshold used to assess a viable blastocyst [17]. Thus, 5 embryos created will, on average, give 2–3 blastocysts for PGT-A selection. This group will therefore exclude some cycles when sufficient blastocysts were created from fewer embryos; however, the nature of the banded data precludes an analysis of cycles with fewer embryos created. It will additionally include a number of cycles which suffered over-stimulation and necessitated a “freeze-all” and delayed transfer.

3. As a sensitivity analysis, we also considered a control group that consisted of cycles with > 5 embryos and an embryo transfer (thus excluding the freeze-alls) defining a control group with: 
  $$\text{Embryos.Created > 5 and Embryos.Transferred > 0 and Fresh}$$

All analyses were conducted in the R statistical environment (v4.1).

### Primary outcome

The primary outcome in the analyses here is a live birth event (LBE) in the specific treatment cycle. The inclusion criteria restrict this to cycles started with an intention to create a baby and the selection of cases and controls to those cycles that have progressed as far as having embryos considered suitable for transfer.

### Outcomes not considered

Ideally, we would have used LBE in the first transfer of a sequence of cycles associated with each egg retrieval, mimicking one of the outcomes in the Cochrane review [9]. Whilst the Register does contain the information necessary to determine this, the publicly released data used here do not.

We would also like to consider cumulative LBE over all the transfers following an egg retrieval. This is potentially derivable from the full Register, but not available in the public dataset. Register data are not yet available beyond 2018, and therefore there is not yet long enough follow-up for cycles commenced in 2018.

Multiple birth outcomes would also form part of a comprehensive evaluation, both multiples per LBE and per cycle.

LBE per embryo transferred is not an appropriate comparison for a treatment option which reduces the number of cycles where embryo transfer takes place.

### Models

We fitted logistic regression models for LBE with the following covariates:

1. PGT-A as a main effect plus age band and the covariates listed below. This provides estimates of the overall effect of PGT-A versus the controls
2. PGT-A, age band *and their interaction* along with the covariates listed below. This model was parametrised to give estimates of the PGT-A effect in each age band.

Model estimates are presented as *OR* with 95% *CI* for the overall effects and 99% *CI* for the individual age bands to account for multiple testing. A likelihood ratio test comparing the two models was used to test the statistical significance of the interaction term — that is, whether the effect of PGT-A varied with age group.

### Covariates included

*Patient characteristics*: age, previous ART cycles (as categorised), previous LB (noting we only have births arising from ART), cause of infertility (5 binary variables). Age is known to be strongly non-linear and an ideal representation should capture the shape of this relationship rather more accurately that the limited age bands.

*Treatment specification*: IVF/ICSI, eSET (which should be pre-specified rather than consequential on the availability of embryos), use of hormonal stimulation (as this will affect the uterine environment).

*Year*: to allow for temporal effects.

### Covariates not included

*Fresh/frozen*: is at least in part related to the use of PGT so should not be included.

*Number of embryos transferred*: is dependent on the embryo selection so should not be included.

### Covariates that could not be included

*Pre-PGT outcomes*: (eggs collected and embryos created) should be included as they are associated with the decision to conduct PGT-A, but are not available in this dataset for frozen cycles which form most of PGT cohort. Also, the public data aggregate those with 1–5 eggs/embryos so lacks the detail at the low end which is necessary to use this as a covariate.

*Embryo stage at transfer*: should be included but not available for most frozen cycles in this dataset, but is potentially available in the Register.

*Centre and funding source*: PGT-A is offered by relatively few centres to self-funded patients. Thus, the PGT-A-treated patients have potentially very different demographics, treatment protocols and pathways along with differing treatment eligibility criteria. Although not in the public dataset, these data are available in the full Register.

*Ethnicity*: This is in the Register but not the public dataset and may be incomplete or poor quality.

*Duration of infertility*: This is in the Register but not the public dataset and may be incomplete or of poor quality.

## Results

### Patient and treatment characteristics

These are shown in Table 1 for the PGT-A and control datasets. The control groups comprise ~ 25% of the non-PGT-A cycles; 32% of the non-PGT-A cycles are the secondary frozen cycles; and 39% have few embryos created and are therefore excluded as controls. PGT-A was recorded in a small number of cycles where fewer than 6 embryos were created. Of the PGT-A cycles, 13% were associated with fresh cycles.

**Table 1:** Characteristics of the PGT-A cycles, non-PGT-A cycles and the two control groups

				**Controls**	
		**PGT-A**	**All non PGT-A**	** > 1 embryo at transfer**	** > 5 embryos created**
**Patient characteristics**					
Age	18–34	455/2192 (20.8%)	81,752/189610 (43.1%)	24,309/44656 (54.4%)	28,069/52863 (53.1%)
	35–37	507/2192 (23.1%)	45,532/189610 (24.0%)	11,215/44656 (25.1%)	12,317/52863 (23.3%)
	38–39	420/2192 (19.2%)	28,177/189610 (14.9%)	5532/44656 (12.4%)	6570/52863 (12.4%)
	40–42	585/2192 (26.7%)	25,424/189610 (13.4%)	3175/44656 (7.1%)	4856/52863 (9.2%)
	43–44	181/2192 (8.3%)	6581/189610 (3.5%)	368/44656 (0.8%)	884/52863 (1.7%)
	45–50	44/2192 (2.0%)	2144/189610 (1.1%)	57/44656 (0.1%)	167/52863 (0.3%)
Diagnosis^1^	Tubal	132/2192 (6.0%)	21,145/189610 (11.2%)	5255/44656 (11.8%)	6075/52863 (11.5%)
	Ovul	259/2192 (11.8%)	24,418/189610 (12.9%)	5696/44656 (12.8%)	7699/52863 (14.6%)
	Endo	78/2192 (3.6%)	11,756/189610 (6.2%)	2780/44656 (6.2%)	3171/52863 (6.0%)
	Male	520/2192 (23.7%)	67,807/189610 (35.8%)	16,075/44656 (36.0%)	19,186/52863 (36.3%)
	Unexplained	862/2192 (39.3%)	60,243/189610 (31.8%)	15,230/44656 (34.1%)	17,545/52863 (33.2%)
Previous IVF treatments	0	99/2192 (4.5%)	75,495/189610 (39.8%)	29,935/44656 (67.0%)	33,232/52863 (62.9%)
	1	449/2192 (20.5%)	50,940/189610 (26.9%)	6648/44656 (14.9%)	8670/52863 (16.4%)
	2	380/2192 (17.3%)	29,045/189610 (15.3%)	3672/44656 (8.2%)	4967/52863 (9.4%)
	3	350/2192 (16.0%)	15,626/189610 (8.2%)	2145/44656 (4.8%)	2814/52863 (5.3%)
	4	281/2192 (12.8%)	8330/189610 (4.4%)	1083/44656 (2.4%)	1476/52863 (2.8%)
	> 4	633/2192 (28.9%)	10,174/189610 (5.4%)	1173/44656 (2.6%)	1704/52863 (3.2%)
**Treatment characteristics**					
Fresh	All	294/2192 (13.4%)	128,292/189610 (67.7%)	44,656/44656 (100.0%)	52,863/52863 (100.0%)
	IVF^2^	105/2192 (4.8%)	61,323/189610 (32.3%)	21,071/44656 (47.2%)	24,725/52863 (46.8%)
	ICSI^2^	186/2192 (8.5%)	66,450/189610 (35.0%)	23,441/44656 (52.5%)	27,725/52863 (52.4%)
	Mixed IVF/ICSI^2^	3/2192 (0.1%)	517/189610 (0.3%)	143/44656 (0.3%)	412/52863 (0.8%)
Frozen	All	1898/2192 (86.6%)	61,318/189610 (32.3%)	0/44656 (0.0%)	0/52863 (0.0%)
Stimulation		296/2192 (13.5%)	126,017/189610 (66.5%)	44,437/44656 (99.5%)	52,694/52863 (99.7%)
Embryos transferred	0	375/2192 (17.1%)	24,094/189610 (12.7%)	0/44656 (0.0%)	5566/52863 (10.5%)
	1	1621/2192 (74.0%)	105,191/189610 (55.5%)	35,038/44656 (78.5%)	30,607/52863 (57.9%)
	2	190/2192 (8.7%)	57,094/189610 (30.1%)	9278/44656 (20.8%)	15,547/52863 (29.4%)
	3	6/2192 (0.3%)	3231/189610 (1.7%)	340/44656 (0.8%)	1143/52863 (2.2%)
eSET		1009/2192 (46.0%)	68,971/189610 (36.4%)	30,642/44656 (68.6%)	26,682/52863 (50.5%)
Transfer stage	Cleavage	27/2192 (1.2%)	39,667/189610 (20.9%)	3531/44656 (7.9%)	4286/52863 (8.1%)
	Blast	166/2192 (7.6%)	71,264/189610 (37.6%)	41,046/44656 (91.9%)	42,929/52863 (81.2%)
	Unknown	1999/2192 (91.2%)	78,679/189610 (41.5%)	79/44656 (0.2%)	5648/52863 (10.7%)
Year treatment started	2016	396/2192 (18.1%)	62,942/189610 (33.2%)	14,593/44656 (32.7%)	18,060/52863 (34.2%)
	2017	816/2192 (37.2%)	63,966/189610 (33.7%)	15,486/44656 (34.7%)	18,094/52863 (34.2%)
	2018	980/2192 (44.7%)	62,702/189610 (33.1%)	14,577/44656 (32.6%)	16,709/52863 (31.6%)
**Treatment response**					
Eggs collected (fresh only)	0	6/294 (2.0%)	7971/128292 (6.2%)	131/44656 (0.3%)	164/52863 (0.3%)
	1–5	40/294 (13.6%)	31,771/128292 (24.8%)	3359/44656 (7.5%)	2/52863 (0.0%)
	6–10	84/294 (28.6%)	40,779/128292 (31.8%)	15,200/44656 (34.0%)	13,815/52863 (26.1%)
	11–15	81/294 (27.6%)	27,143/128292 (21.2%)	14,518/44656 (32.5%)	20,255/52863 (38.3%)
	16–20	57/294 (19.4%)	12,659/128292 (9.9%)	7597/44656 (17.0%)	11,147/52863 (21.1%)
	21–25	14/294 (4.8%)	4930/128292 (3.8%)	2711/44656 (6.1%)	4590/52863 (8.7%)
	26–30	8/294 (2.7%)	1898/128292 (1.5%)	800/44656 (1.8%)	1804/52863 (3.4%)
	31–35	3/294 (1.0%)	680/128292 (0.5%)	224/44656 (0.5%)	644/52863 (1.2%)
	36–40	0/294 (0.0%)	263/128292 (0.2%)	70/44656 (0.2%)	252/52863 (0.5%)
	> 40	1/294 (0.3%)	198/128292 (0.2%)	46/44656 (0.1%)	190/52863 (0.4%)
Embryos created (fresh only)	0	1/294 (0.3%)^3^	13,667/128292 (10.7%)	0/44656 (0.0%)	0/52863 (0.0%)
	1–5	97/294 (33.0%)	61,762/128292 (48.1%)	12,731/44656 (28.5%)	0/52863 (0.0%)
	6–10	118/294 (40.1%)	37,069/128292 (28.9%)	21,717/44656 (48.6%)	37,069/52863 (70.1%)
	11–15	62/294 (21.1%)	11,872/128292 (9.3%)	8085/44656 (18.1%)	11,872/52863 (22.5%)
	16–20	13/294 (4.4%)	2952/128292 (2.3%)	1761/44656 (3.9%)	2952/52863 (5.6%)
	21–25	2/294 (0.7%)	716/128292 (0.6%)	288/44656 (0.6%)	716/52863 (1.4%)
	26–30	1/294 (0.3%)	184/128292 (0.1%)	60/44656 (0.1%)	184/52863 (0.3%)
	> 40	0/294 (0.0%)	70/128292 (0.1%)	14/44656 (0.0%)	70/52863 (0.1%)
Embryos stored (fresh only)	0	107/294 (36.4%)	76,842/128292 (59.9%)	0/44656 (0.0%)	15,722/52863 (29.7%)
	1–5	159/294 (54.1%)	43,695/128292 (34.1%)	39,834/44656 (89.2%)	29,397/52863 (55.6%)
	6–10	27/294 (9.2%)	6419/128292 (5.0%)	4429/44656 (9.9%)	6411/52863 (12.1%)
	11–15	1/294 (0.3%)	982/128292 (0.8%)	347/44656 (0.8%)	980/52863 (1.9%)
	16–20	0/294 (0.0%)	249/128292 (0.2%)	36/44656 (0.1%)	248/52863 (0.5%)
	> 20	0/294 (0.0%)	105/128292 (0.1%)	10/44656 (0.0%)	105/52863 (0.2%)
Live birth		809/2192 (36.9%)	52,336/189610 (27.6%)	19,476/44656 (43.6%)	18,993/52863 (35.9%)
Multiple birth		48/2192 (2.2%)	5222/189610 (2.8%)	1613/44656 (3.6%)	1969/52863 (3.7%)

^1^Zero, one or more diagnoses may be recorded for each couple

^2^There were two cycles with missing data for IVF/ICSI

^3^Must be a data error as no biopsy could be performed if no embryos created

### PGT-A v non-PGT-A

Comparing PGT-A with all non-PGT-A cycles, the dataset used here replicates the results derived from the FoI dataset (Supplementary Table S2). It is worth noting that the limited covariate adjustment does not make a big difference to the estimates, although as discussed above, many important covariates are not available in the public dataset.

### PGT-A v controls

Taking a plausibly appropriate control group of cycles which could have had PGT-A, we see that the treatment effect of PGT-A is markedly different with overall *OR* for LBE of 0.82 (0.68–1.00) using the > 1 transferrable embryo controls (Table 2) and 0.80 (0.64–0.99) using the > 5 embryos created controls (Table 3), both suggesting that PGT-A has a negative effect on LBE. The estimates are compatible with the estimates from the recent randomised trials.

**Table 2:** Comparison of PGT-A with a control group defined as having > 1 embryo at transfer

Age	Controls	PGT-A	*OR*^a^	*OR*_adj_^b^
18–34	11,766/24309 (48.4%)	182/455 (40.0%)	0.71 (0.55–0.91)	0.53 (0.38–0.74)
35–37	4809/11215 (42.9%)	209/507 (41.2%)	0.93 (0.74–1.18)	0.71 (0.51–0.99)
38–39	1942/5532 (35.1%)	172/420 (41.0%)	1.28 (0.98–1.67)	0.99 (0.70–1.40)
40–42	910/3175 (28.7%)	199/585 (34.0%)	1.28 (1.00–1.64)	1.01 (0.72–1.41)
43–44	42/368 (11.4%)	40/181 (22.1%)	2.20 (1.18–4.12)	1.82 (0.95–3.49)
45–50	7/57 (12.3%)	7/44 (15.9%)	1.35 (0.31–5.97)	1.15 (0.26–5.13)
**Overall**	**19,476/44656 (43.6%)**	**809/2192 (36.9%)**	**0.76 (0.69–0.83)**	**0.82 (0.68–1.00)**
Age by PCT-A interaction *P*				< 0.001

Odds ratios ^a^ without and ^b^ with covariate adjustment and 95% CI for the overall effect and 99% CI for the individual age bands

**Table 3:** Comparison of PGT-A with a control group defined as having > 5 embryos created

Age	Controls	PGT-A	*OR*^a^	*OR*_adj_^b^
18–34	11,184/28069 (39.8%)	182/455 (40.0%)	1.01 (0.79–1.29)	0.62 (0.43–0.89)
35–37	4573/12317 (37.1%)	209/507 (41.2%)	1.19 (0.94–1.51)	0.70 (0.49–1.00)
38–39	2018/6570 (30.7%)	172/420 (41.0%)	1.56 (1.20–2.04)	0.89 (0.61–1.28)
40–42	1110/4856 (22.9%)	199/585 (34.0%)	1.74 (1.37–2.21)	0.90 (0.63–1.28)
43–44	91/884 (10.3%)	40/181 (22.1%)	2.47 (1.44–4.25)	1.38 (0.77–2.50)
45–50	17/167 (10.2%)	7/44 (15.9%)	1.67 (0.48–5.82)	0.98 (0.27–3.53)
**Overall**	**18,993/52863 (35.9%)**	**809/2192 (36.9%)**	**1.04 (0.95–1.14)**	**0.80 (0.64–0.99)**
Age by PCT-A interaction *P*				0.003

Odds ratios ^a^ without and ^b^ with covariate adjustment and 95% CI for the overall effect and 99% CI for the individual age bands

There is evidence of an age by PGT-A interaction with PGT-A being less disadvantageous in older women. However, there are few of these in the dataset and the selection biases are very strong in this age group where NHS treatment is not available, so this has to be treated very cautiously.

A sensitivity analysis with a more tightly defined control group shows similar results (Supplementary Table S3).

## Discussion

The estimates derived here demonstrate clearly that the use of crude aggregated national data which fails to account for confounding and treatment selection effects [16] is highly misleading. The analyses here demonstrate clearly that the conclusions can be reversed by selecting controls more carefully and making a clinically relevant comparison. We must stress that the analysis presented here is not intended to be definitive as the public data have too few data on some important confounders and do not allow consideration of the multiple cycles that comprise a full ART treatment. There is a suggestion (as in some of the trials e.g. [10]) that its efficacy in the first transfer increases with age which may be worthy of further study and further targeted RCTs.

Based on our experience with the public dataset, we can begin to define what an epidemiologically sound analysis to address the effectiveness of PGT-A using the HFEA register would require. This would require a bespoke dataset from the register with a more complete covariate set (as discussed above), linking of cycles relating to the same egg batch and the same woman and information on type of PGT-A.

The analysis would then include the following steps:

1. A careful data cleaning/validation, particularly of the linking of PGT-A indicators across the individual treatment courses.
2. Linking the frozen cycles to the fresh cycle providing the embryos, so enabling covariates relating to egg and embryo number for frozen as well as fresh cycles.
3. Defining and creating multi-cycle outcomes (first transfer LBE, cumulative LBE, time to LBE).
4. Careful consideration of the covariates as discussed above and their representation.
5. Selection of appropriate control groups so that, as far as possible, cycles not eligible for PGT-A are excluded.
6. Application of the same exclusions to the PGT-A as to the control cycles.
7. Fitting appropriate models to estimate the treatment effects adjusting for the covariates.
8. Sensitivity analyses around the exclusions and control group definitions.

However, such an analysis will still have significant weaknesses:

1. There is lack of intermediate outcomes in the Register. It is not possible to rigorously determine those cycles that potentially could get PGT-A and are reliant on surrogates such as the number of embryos created.
2. The HFEA record instances of embryo biopsy rather than the intent to perform embryo biopsy, so will miss cycles where PGT-A was intended, but no eggs were retrieved, or no embryos were available for biopsy.
3. Observational data will always be unreliable when the treatment is determined by patient choice/ability to pay as there are numerous unmeasured and unmeasurable factors that influence this decision, and these could be correlated with prognosis.
4. Whilst we can adjust for measured covariates, these effects are strong [18], and the covariates often are imprecisely recorded. Any such analysis is therefore unlikely to reduce the bias in the treatment *OR* below ± 0.2 which is a substantial treatment effect and about the magnitude any treatment add-on could be likely to achieve (~ 5% or so absolute uplift in LBE).
5. Any bespoke dataset will exclude those patients who did not give their consent for data disclosure. Data from another study suggests that this could amount to a loss of 40% of the PGT-A cycles and 35% of non-PGT-A cycles in the 2016–2018 timeframe with correlation between clinics with poor consent rates and those offering PGT-A. This reduces the power and precision of the estimates and potentially leads to significant biases in any estimates.
6. Larger, more detailed and more recent data would give the power to look at sub-types of PGT-A and to look specifically at the more recent iterations of PGT-A. However, data beyond 2018 are not likely to be available before mid-2023 due to the migration of database systems within the HFEA. The COVID-19 pandemic led to the temporary closure of many clinics, so data for 2020/21 are limited.

It is therefore not clear whether the biases and limitations inherent in the Registry data will allow useful conclusions to be drawn (see also [19]). We are currently going through the design and approval process to obtain a bespoke dataset to allow us to assess what can be achieved.

## Conclusion

We have shown that, if we compare like with like, we obtain estimates of the effect of PGT-A from the publicly available HFEA Register data that suggest an overall modest reduction in LBE. These are in direct contrast to the ill-founded claims made by others [15, 16]. A detailed analysis of a fuller dataset is warranted, but it remains to be demonstrated whether the UK Register data can provide useful estimates of the utility of PGT-A as a treatment add-on.

## Supplementary Information

Below is the link to the electronic supplementary material.

Supplementary file1 (DOCX 20 KB)
