# Supplementary material for: Does PGT-A improve assisted reproduction treatment success rates: what can the UK Register data tell us?
Source: J Assist Reprod Genet. 2022 Sep 21;39(11):2547–54. doi: 10.1007/s10815-022-02612-y (PMC9490705; doi:10.1007/s10815-022-02612-y)
Supplement: Supplementary file 1 — Supplementary file1 (DOCX 20 KB) [file 10815_2022_2612_MOESM1_ESM.docx]

# Supplementary Material: Does PGT-A improve Assisted Reproduction Treatment success rates: What can the UK Register data tell us?

**Table S1:** The entirety of the data provided by the HFEA under the FoI request

| **Treatment type** | **Patient age** | **Cycles where no embryos transferred** | **Cycles where at least one embryo transferred** | **Total Cycles** | **Embryos Transferred** | **Live Birth Occurrence** | **Live Birth rate PET** | **Live Birth rate PTC** |
| --- | --- | --- | --- | --- | --- | --- | --- | --- |
| **IVF (excl. PGD & PGS)** | **Under 35** | 9,753 | 71,218 | 80,971 | 90,097 | 27,449 | 30.50% | 33.90% |
|  | **35-37** | 5,021 | 39,993 | 45,014 | 53,394 | 13,174 | 24.70% | 29.27% |
|  | **38-39** | 3,581 | 24,314 | 27,895 | 35,560 | 6,388 | 18.00% | 22.90% |
|  | **40-42** | 3,876 | 21,226 | 25,102 | 35,484 | 3,887 | 11.00% | 15.48% |
|  | **43-44** | 1,279 | 5,192 | 6,471 | 9,190 | 421 | 4.60% | 6.51% |
|  | **Over 44** | 578 | 1,515 | 2,093 | 2,524 | 72 | 2.90% | 3.44% |
|  | **All** | 24,088 | 163,458 | 187,546 | 226,249 | 51,391 | 22.71% | 27.40% |
| **PGS (Fresh & frozen)** | **Under 35** | 68 | 459 | 527 | 529 | 203 | 38.40% | 38.52% |
|  | **35-37** | 73 | 508 | 581 | 551 | 238 | 43.20% | 40.96% |
|  | **38-39** | 69 | 393 | 462 | 436 | 183 | 42.00% | 39.61% |
|  | **40-42** | 118 | 536 | 654 | 578 | 219 | 37.90% | 33.49% |
|  | **43-44** | 69 | 129 | 198 | 144 | 43 | 29.90% | 21.72% |
|  | **Over 44** | 21 | 21 | 42 | 22 | 7 | 31.80% | 16.67% |
|  | **All** | 418 | 2,046 | 2,464 | 2,260 | 893 | 39.51% | 36.24% |

**Table S2:** Results from the public register comparing all PGT-A with all non-PGT-A cycles.

| **Age** | **All non-PGT-A** | **PGT-A** | **OR ^a^** | **OR_adj_ ^b^** |
| --- | --- | --- | --- | --- |
| 18-34 | 27869/81755 (34.1%) | 182/455 (40.0%) | 1.29 (1.01-1.65) | 1.19 (0.93-1.53) |
| 35-37 | 13420/45539 (29.5%) | 209/507 (41.2%) | 1.68 (1.33-2.12) | 1.48 (1.17-1.88) |
| 38-39 | 6514/28186 (23.1%) | 172/421 (40.9%) | 2.30 (1.78-2.97) | 1.95 (1.50-2.54) |
| 40-42 | 4025/25441 (15.8%) | 199/585 (34.0%) | 2.74 (2.18-3.45) | 2.23 (1.77-2.81) |
| 43-44 | 438/6590 (6.6%) | 40/182 (22.0%) | 3.96 (2.45-6.38) | 3.34 (2.06-5.41) |
| 45-50 | 82/2152 (3.8%) | 7/44 (15.9%) | 4.78 (1.59-14.36) | 4.16 (1.38-12.56) |
| **Overall** | 52348/189663 (27.6%) | 809/2194 (36.9%) | 1.53 (1.40-1.67) | **1.74 (1.59-1.91)** |
|  |  | Age by PCT-A interaction P | | <0.001 |

Odds ratios ^a^ without and ^b^ with covariate adjustment and 95% CI for the overall effect and 99% CI for the individual age bands

**Table S3**: Sensitivity analysis: PGT-A v Controls with >5 embryos created and an embryo transfer

| **Age** | **Controls** | **PGT-A** | **OR ^a^** | **OR_adj_ ^b^** |
| --- | --- | --- | --- | --- |
| 18-34 | 11179/24469 (45.7%) | 182/455 (40.0%) | 0.79 (0.62-1.02) | 0.48 (0.34-0.69) |
| 35-37 | 4569/11256 (40.6%) | 209/507 (41.2%) | 1.03 (0.81-1.30) | 0.62 (0.44-0.88) |
| 38-39 | 2017/6074 (33.2%) | 172/420 (41.0%) | 1.40 (1.07-1.82) | 0.84 (0.58-1.21) |
| 40-42 | 1110/4515 (24.6%) | 199/585 (34.0%) | 1.58 (1.24-2.01) | 0.93 (0.66-1.32) |
| 43-44 | 91/824 (11.0%) | 40/181 (22.1%) | 2.29 (1.33-3.93) | 1.47 (0.82-2.64) |
| 45-50 | 17/159 (10.7%) | 7/44 (15.9%) | 1.58 (0.45-5.52) | 0.99 (0.28-3.55) |
| **Overall** | 18983/47297 (40.1%) | 809/2192 (36.9%) | 0.87 (0.80-0.95) | **0.73 (0.59-0.90)** |
|  | Age by PCT-A interaction P | | | <0.001 |

Odds ratios ^a^ without and ^b^ with covariate adjustment and 95% CI for the overall effect and 99% CI for the individual age bands
